# A 14 nucleotide deletion mutation in the coding region of the *PpBBX24* gene is associated with the red skin of “Zaosu Red” pear (*Pyrus pyrifolia* White Pear Group): a deletion in the *PpBBX24* gene is associated with the red skin of pear

**DOI:** 10.1038/s41438-020-0259-7

**Published:** 2020-04-01

**Authors:** Chunqing Ou, Xiaoli Zhang, Fei Wang, Liyi Zhang, Yanjie Zhang, Ming Fang, Jiahong Wang, Jixun Wang, Shuling Jiang, Zhihong Zhang

**Affiliations:** 1 College of Horticulture, Shenyang Agricultural University, Shenyang, 110161 Liaoning PR China; 2 Key Laboratory of Horticultural Crops Germplasm Resources Utilization, Ministry of Agriculture, Research Institute of Pomology, Chinese Academy of Agricultural Sciences, Xingcheng, 125100 Liaoning PR China; 3 Xinjiang Fruit Science Experiment Station, Ministry of Agriculture and Rural Affairs, Horticultural Crops Research Institute, Xinjiang Academy of Agricultural Sciences, Urumqi, 830091 Xinjiang PR China; 4 Biomarker Technologies Corporation, Beijing, 101300 PR China

**Keywords:** Plant molecular biology, Natural variation in plants, Plant hybridization

## Abstract

Red skin is an important quality trait for pear fruits and is determined by the concentration and composition of anthocyanins. The regulatory mechanism underlying anthocyanin accumulation is a popular topic in fruit research. Red mutants are ideal materials for studying the molecular mechanism of color diversity in pear. Although several red pear mutants have been cultivated and are in production, no exact locus containing the responsible genetic mutation has been identified. In this study, by combining the bulked segregant analysis with whole-genome sequencing, we identified a 14 nucleotide deletion mutation in the coding region of the *PpBBX24* gene from the red pear mutant “Zaosu Red”. We further verified that the deletion was present only in the red mutant of “Zaosu” and in its red offspring, which was different from that which occurred in other red pear fruits. This deletion results in a coding frame shift such that there is an early termination of the *PpBBX24* gene and loss of key NLS and VP domains from PpBBX24. The lost domains may reduce or alter the normal function of PpBBX24. In addition, we found that the transcript levels of the *PpMYB10* and *PpHY5* genes in red samples were significantly higher than those in green samples, whereas the results for the normal-type *PpBBX24* gene were the opposite. We ultimately revealed that the 14 nucleotide deletion mutation in the coding region of the *PpBBX24* gene is associated with the red skin of the “Zaosu Red” pear. This finding of somatic mutational events will be helpful for breeding new red pear cultivars and for understanding the regulatory mechanisms involved in pear skin pigmentation.

## Introduction

Pear (*Pyrus* spp.) fruits are very popular and are widely grown in temperate regions all worldwide^1^. The red skin of pear is one of the most preferred preferences to consumers because of its attractive appearance and nutritional value^2^, and a study in Australia and New Zealand showed that a large percentage of consumers were willing to buy a novel red-skinned pear despite its poor flavor^3^. In pear production, red pear cultivars are common for occidental pear (*Pyrus communis* L.). For oriental pear, especially Chinese sand pear (*P. pyrifolia* Nakai) and Chinese white pear (*P. pyrifolia* White Pear Group), red peel color is hard to obtain and is unstable in different regions and under different cultivation conditions^4–6^; fully red-skinned cultivars are rare. Previous studies have shown that the developmental patterns of red coloration are different among plants and pear cultivation groups^1,7,8^, and it has been shown that the red color is a dominant characteristic and that the ratio of red-skinned fruits in progeny is 1:1 for the crosses between a yellow pear parent and heterozygous red pear parent^9,10^.

The concentration and composition of anthocyanins are the main determinants of red coloration of pear fruits; these determinants are regulated by many genes and are affected by various environmental conditions, including light and temperature^2,5,11–14^. Previous studies have shown that the components of anthocyanins in red-skinned pears mainly include cyanidin-3-galactoside (C-Ga), peonidin-3-galactoside (P-Ga), cyanidin-3-glucoside, cyanidin-3-arabinofuranoside, and peonidin-3-glucoside^15^. The anthocyanin biosynthetic pathway and its regulatory mechanisms have been well elucidated at the transcriptional level in many plant species. Phenylalanine ammonia-lyase (PAL), chalcone synthase (CHS), chalcone isomerase (CHI), flavanone-3-hydroxylase (F3H), dihydroflavonol 4-reductase, anthocyanidin synthase (ANS), and UDP-glucose: flavonoid 3-glucosyltransferase (UFGT) are the key enzymes involved in anthocyanin biosynthesis in pear and other plant species^16–18^.

Furthermore, there are numerous reports of transcription factor proteins in the anthocyanin biosynthesis pathway. Among these proteins, R2R3-type MYBs are the most important for anthocyanin accumulation^19,20^, as they regulate the expression of anthocyanin biosynthesis genes by forming MYB–bHLH–WD40 (MBW) protein complexes combined with basic helix–loop–helix (bHLH) and W40 repeat proteins^6,21^. In pear, PcMYB10 and PyMYB114 have been verified to have such functions^22,23^.

In addition to their involvement in MBW complexes, other transcription factors also have important effects on anthocyanin biosynthesis by interacting with the complex. Recently, Tao et al.^24^ revealed that *PpHY5* participated in anthocyanin biosynthesis induced by blue light in red pear. Bai et al.^6,25^ found that PpBBX16 cooperated with PpHY5 and positively regulated light-induced anthocyanin accumulation by activating MYB10 in red pear; PpBBX18 and PpBBX21 antagonistically regulate anthocyanin biosynthesis via competitive association with PpHY5 in the peel of pear fruits. Ni et al.^14^ reported that the ethylene response factors Pp4ERF24 and Pp12ERF96 regulate blue light-induced anthocyanin biosynthesis in red pear fruits by interacting with MYB114, and Wu et al.^26^ indicated that *PyMADS18* is likely to be involved in anthocyanin accumulation and the regulation of anthocyanin synthesis in the early development of pear fruits. Thus, the regulatory networks of anthocyanin biosynthesis are intricate and complex.

**Fig. 1:**
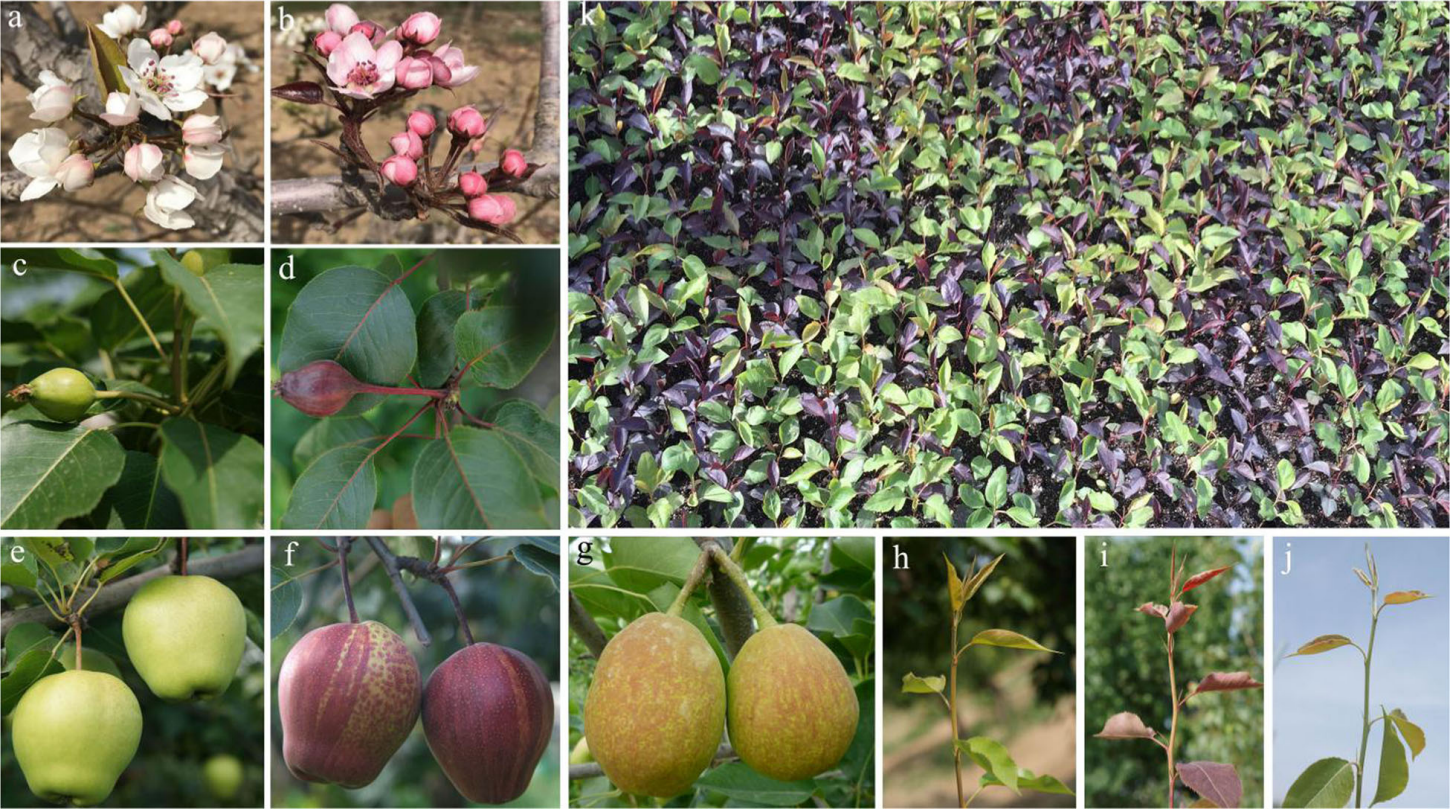
Flowers, fruits, new shoots, and seedlings of “Zaosu Red”, “Zaosu”, and “Kuala Pear”. **a**, **c**, **e**, and **h** respectively show flowers, young fruits, mature fruits, and new shoots of “Zaosu”; **b**, **d**, **f**, and **i** respectively show flowers, young fruits, mature fruits, and new shoots of “Zaosu Red”; **g** and **j** respectively show fruits and new shoots of “Kuala Pear”; **k** shows seedlings of “Kuala Pear” × “Zaosu Red”

“Zaosu” is a pear cultivar widely cultivated in China and has crisp, green-skinned fruit (Fig. 1e); this cultivar is a hybrid of “Pingguoli” (female parent, *P. pyrifolia* White Pear Group) and “Shenbuzhi” (male parent, *P. communis*). “Zaosu Red,” whose phenotype, including that of its fruits, flowers, stems, and young leaves, is red, originated from the discovery of a red mutant of “Zaosu” (Fig. 1a–f, h, i). The mutant inherits the excellent fruit quality and cultivation traits of “Zaosu” and is an exceptional parent for breeding new varieties with crisp, red-skinned fruits. Because its genetic background is nearly identical to that of its original type, “Zaosu Red” is also an ideal material to study the molecular mechanism of color diversity in pear. Although recent studies have demonstrated that the genetic locus underlying red foliage and fruit skin traits had been mapped to LG4 in “Red Zaosu”^27^, the exact locus of the genetic mutation remains elusive.

In this study, by combining bulked segregant analysis (BSA) with whole-genome sequencing, we identified a 14 nucleotide deletion that is located in the coding region of the *PpBBX24* gene and that is associated with the red color of “Zaosu Red”. This finding of somatic mutational events will be helpful for breeding new red pear cultivars and understanding the regulatory mechanisms involved in pear skin pigmentation.

## Results

### Anthocyanin content in the leaves and fruit peels of pear

**Fig. 2:**
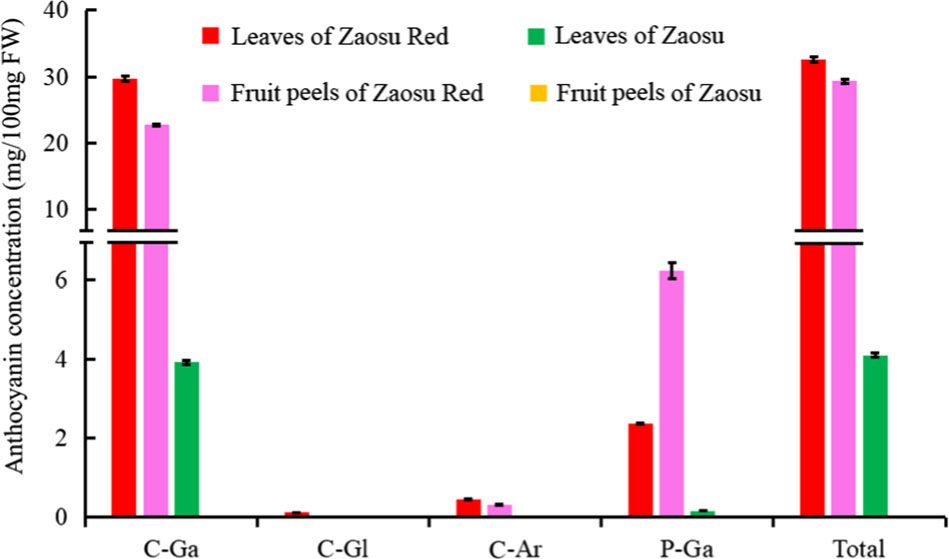
Anthocyanin concentrations in different samples, as detected by UPLC-PDA-MS/MS. C-Ga, C-Gl, C-Ar, and P-Ga represent cyanidin-3-galactoside, cyanidin-3-O-glucoside, cyanidin-3-O-arabinoside, and peonidin-3-O-galactoside, respectively. Total represents the sum of all the above anthocyanins

As shown in Fig. 2, four known anthocyanins were detected in the leaves and peels of young fruits of “Zaosu Red”, and the contents in “Zaosu Red” were significantly higher than those in “Zaosu”. No anthocyanins were detected in the peels of young fruits of “Zaosu”, and only a few C-Ga and P-Ga were detected in the leaves of “Zaosu”. Among the four anthocyanins, C-Ga constituted the majority (77.7–95.8%), P-Ga was the next most abundant (4.2–21.2%), and the other two were present at very low levels (<2%). Of the major two kinds of anthocyanins in “Zaosu Red”, the proportions between the leaves and fruits were different. The proportion of C-Ga in the leaves was 91.0%, which was higher than that in fruits (77.7%), while the proportion of P-Ga in the leaves was 7.3%, which was lower than that in fruits (21.2%).

### Whole-genome resequencing analysis

Here, five leaf samples from two parents (“Zaosu Red” and “Kuala Pear”), two pools (red and green), and “Zaosu” were sequenced. A total of 96.91 Gb of clean bases were obtained, with an average coverage depth of 24.64× the reference genome among two parents and “Zaosu” and 57.80× coverage between the two pools (the reference genome was estimated to be ~511.33 Mb). Approximately 80% of these reads were properly mapped to the reference genome, and they were used for further analysis (Supplementary Table S1). The clean read data have been deposited in the NCBI Sequence Read Archive (SRA) database under accession number SRP225824.

### SNP/InDel variant detection and annotation

To obtain accurate variants, the single-nucleotide polymorphism (SNP)/InDel variants were filtered through a series of steps after detection by GATK. In total, 4.86 (“Kuala Pear”) to 6.90 (red pool) million SNPs and 0.92 (“Kuala Pear”) to 1.27 (red pool) million InDels were detected against the reference genome from the test samples; among these data, the frequency of the SNPs was more than five times that of the InDels, the frequency of the transition-type SNPs was approximately two times that of the transversion types, the frequency of insertions was slightly less than that of deletions, and the heterozygosity ratios of SNPs and the homozygosity ratios of InDels were more than 60% in all the test samples (Supplementary Table S2). According to the annotation information, ~30% of the SNPs/InDels were located in the intergenic region, ~40% were in upstream and downstream of genes, ~10% were in introns, and only ~6% of SNPs and 1.5% of InDels were located in the coding sequence (CDS) region in every test sample. Approximately 53 and 45% of the SNPs in the CDS region caused synonymous coding and nonsynonymous coding variants, respectively. Approximately 54% of the InDels in the CDS region caused frame shift variants (Supplementary Tables S3–S6).

### Association analysis

To obtain high-quality variants, before association analysis, the SNP/InDel variants that had multiple genotypes, which were supported by less than four reads, whose genotypes are the same in both pools and which are not from the recessive parent in the recessive pool, were filtered, and a total of 731,881 high-quality SNPs and 470,493 high-quality InDels were ultimately obtained (Supplementary Table S7). The euclidean distance (ED) and SNP index values of all the high-quality SNPs/InDels were then calculated and fitted using a sliding window approach with 2 Mb windows sliding in 10 kb steps.

**Fig. 3:**
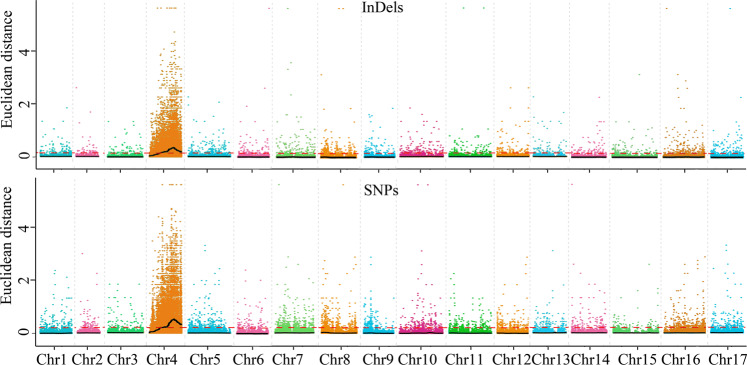
ED value distribution of InDels and SNPs in chromosomes. The abscissa represents the chromosome name, the colored dots represent ED values, the black lines represent the fitted ED values (with 2 Mb windows sliding in 10 kb steps), and the red dotted lines represent associated thresholds

For the ED method, the median values +3 SDs (standard deviations) were used as the associated thresholds, which were 0.21 and 0.14 for the SNP and InDel variants, respectively. Only the regions whose fitted SNP and InDel ED values were higher than the corresponding associated thresholds were defined as candidate-associated regions. By the use of this method, a region of Chr 4 from 7.41 to 22.59 Mb was screened, and the highest associated peak appeared around the region of 17.3 Mb. The results of the SNPs and InDels were consistent (Table 1 and Fig. 3).

**Table 1:** Summary of the candidate-associated regions

Method	Variant type	Chromosome ID	Start (bp)	End (bp)	Size (Mb)	Gene number
ED	SNP	Chr 4	7,410,000	7,490,000	0.08	5
		Chr 4	8,080,000	8,780,000	0.7	57
		Chr 4	8,840,000	22,590,000	13.75	1097
	InDel	Chr 4	7,450,000	22,580,000	15.13	1211
SNP index	SNP	Chr 4	60,000	90,000	0.03	6
		Chr 4	6,480,000	6,520,000	0.04	4
		Chr 4	6,720,000	6,730,000	0.01	1
		Chr 4	6,850,000	6,910,000	0.06	4
		Chr 4	6,930,000	11,000,000	4.07	310
	InDel	Chr 4	1	170,000	0.17	14
		Chr 4	10,070,000	10,100,000	0.03	1
		Chr 4	10,310,000	10,380,000	0.07	5
		Chr 4	10,470,000	10,470,000	0	1
		Chr 4	10,490,000	10,990,000	0.5	30
		Chr 4	7,060,000	7,140,000	0.08	6
		Chr 4	7,240,000	7,250,000	0.01	2
		Chr 4	7,300,000	9,390,000	2.09	163
		Chr 4	9,430,000	9,440,000	0.01	1
		Chr 4	9,490,000	9,500,000	0.01	1
		Chr 4	9,560,000	10,000,000	0.44	29
		Chr 11	1,240,000	1,260,000	0.02	4
		Chr 11	520,000	1,220,000	0.7	102

For the SNP index method, regardless of the SNPs and InDels, no region was associated when the confidence level was set to 0.90, so we used the 99th percentiles of the fitted ΔSNP index values as the associated threshold, which were 0.14 and 0.06 for SNP and InDel variants, respectively. By the use of this method, six regions of Chr 4 from 1 bp to 11 Mb and two regions of Chr 11 from 0.52 Mb to 1.26 Mb were screened, in which the region of Chr 4 at ~8.2 Mb was associated with the highest peak (Table 1 and Supplementary Fig. S1).

### Identification of the gene associated with red/green color

The results of the association analysis showed that the main associated regions were located on Chr 4, which contains more than one thousand genes (Table 1, Fig. 3, and Supplementary Fig. S1). Therefore, it is hard to define which gene is truly associated with the characteristic of red/green color. Because “Zaosu Red” is a red mutant of “Zaosu”, we further identified the different SNPs/InDels of Chr 4 between “Zaosu Red” and “Zaosu” and cross-referenced that with the data between “Zaosu Red” and “Kuala Pear”. The results showed that there were 252 and 150 mutually different SNPs and InDels between “Zaosu Red” vs. “Zaosu” and “Zaosu Red” vs. “Kuala Pear”, respectively, in the candidate-associated regions of Chr 4. Of these, five SNPs and eight InDels were heterozygous in both “Zaosu Red” and the red pool and homozygous in “Kuala Pear”, “Zaosu”, and the green pool, respectively (Supplementary Table S8).

Of the remaining five SNPs and eight InDels, only one deletion of 14 nucleotides within a gene produced a coding frame shift, and the gene was annotated as a CO-like (COL) domain-class transcription factor and subsequently named *PpBBX24*, while all the other SNPs and InDels were located in intergenic regions, unknown regions, or in upstream or downstream genes, which did not alter the CDS (Table 2). In addition, this deletion was located at 18,388,296 bp of Chr 4, which was near the highest associated peak region (17.3 Mb) calculated by the ED method. Thus, the *PpBBX24* gene, which contains the 14 nucleotide deletion, was selected as the final candidate gene associated with red/green color for further analysis.

**Table 2:** Location, annotation, and nucleotide types of selected SNPs/InDels in different samples

Variant type	Position	Reference nucleotides	Alternate nucleotides	Zaosu Red	Zaosu	Kuala Pear	Green pool	Red pool	Effect	Gene ID	Nr annotation
SNPs	9,115,802	T	A	0.5	N	7.0	11.0	7.6	Upstream	Pdr4g010210	Adenylate kinase 1, chloroplastic-like
	9,212,167	C	T	0.1	N	2.0	6.0	5.4	Upstream	Pdr4g010260	Zinc finger CCCH domain-containing protein 11-like
	9,212,608	G	T	0.8	N	7.0	15.0	10.7	Upstream	Pdr4g010260	
	9,214,082	T	C	0.3	N	8.0	9.0	12.1	Upstream	Pdr4g010260	
	9,214,271	A	G	0.4	N	8.0	11.0	12.2	Upstream	Pdr4g010260	
InDels	8,553,091	CTT	C	2.4	0.3	0.2	0.6	6.7	Intergenic		
	9,121,649	GT	G	5.4	5.0	17.0	28.0	19.4	Intergenic		
	9,122,408	G	GA	11.4	8.0	7.0	20.0	20.8	Intergenic		
	9,178,944	TAGA	T	2.5	2.0	3.0	5.0	6.1	Unknown		
	9,222,351	T	TA	11.4	8.0	11.0	27.0	14.5	Downstream	Pdr4g010260	Zinc finger CCCH domain-containing protein 11-like
	9,222,379	G	GT	12,4	11,0	12,0	28,0	17,6	Downstream	Pdr4g010260	
	18,388,296	AGCAGCTGAAGTTCC	A	10,5	14,0	20,0	39,0	24,9	Frame shift	Pdr4g016570	COL domain-class transcription factor
	16,860,274	AG	A	18,9	9,0	7,0	13,0	17,7	Downstream	Pdr4g015130	COP9 signalosome complex subunit 7-like

The numbers in the columns represent the supported read depths of reference nucleotides. Alternate nucleotides are separated by commas, and N represents no read support.

### Variant verification, cloning, and bioinformatic analysis of the *PpBBX24* gene

**Fig. 4:**
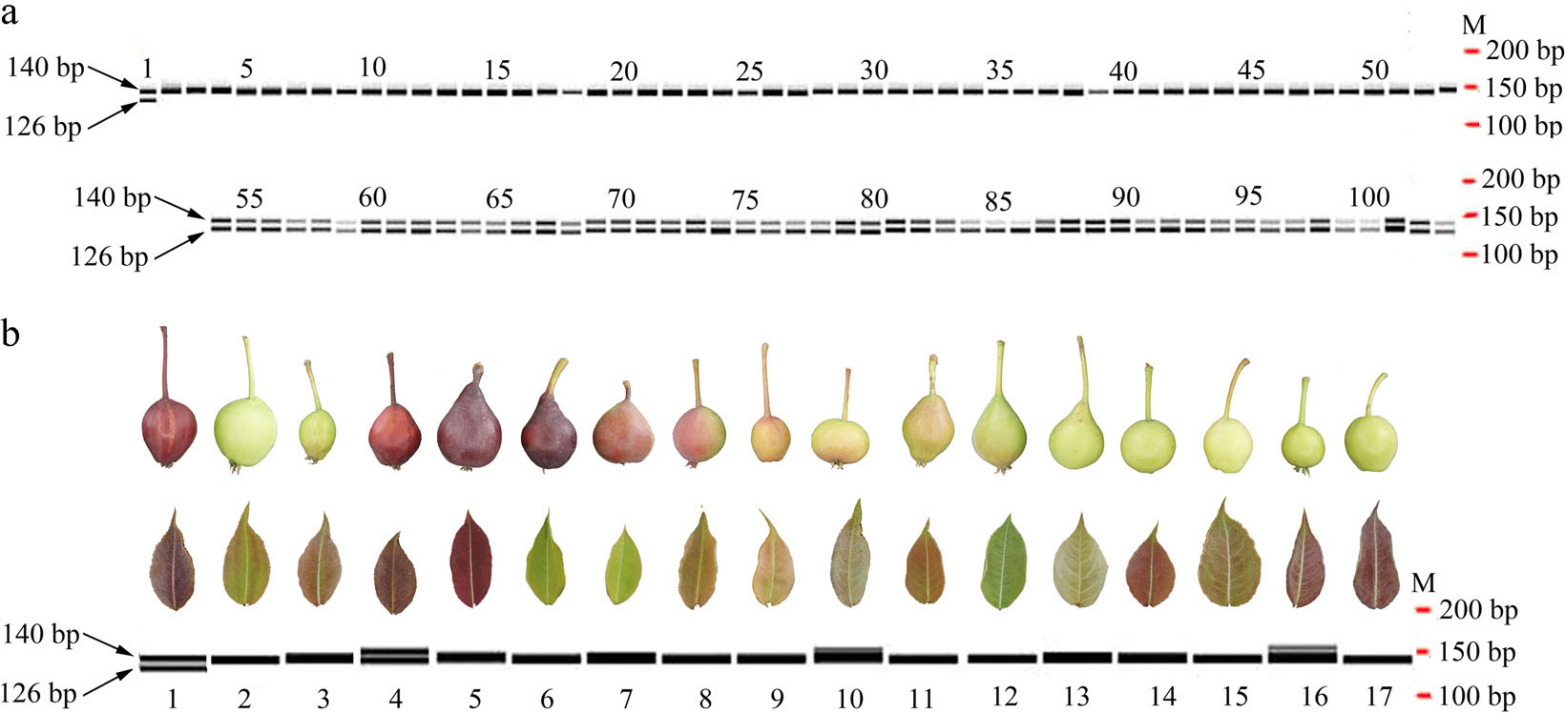
PCR amplification results of the *PpBBX24* gene fragment using the primer pair F-2655 and R-2794 in different DNA samples. **a** PCR amplification results of the leaves of “Zaosu Red”, “Zaosu”, “Kuala Pear”, and green and red F_1_ plants. The maps were generated using LabChip GX Reviewer v5.4 (Perkin-Elmer, USA). M represents “Marker”, and the numbers 1, 2, 3, 4–53, and 54–103 above the bands represent “Zaosu Red”, “Zaosu”, “Kuala Pear”, green F_1_ plants, and red F_1_ plants, respectively. **b** PCR amplification results of the leaves of 18 different cultivars/strains. The band maps were generated using LabChip GX Reviewer v5.4. The numbers below the bands from 1 to 17 represent “Zaosu Red”, “Zaosu”, “Kuala Pear”, “N3-1”, “Red Anjou”, “Red Comice”, “Zhongaihongli”, “Bayuehong”, “Wenshanghongli”, “Pingguoli”, “Jinxiang”, “Zaojinxiang”, “4X Yali”, “Huangguan”, “Dangshansu”, “Jinfeng”, and “E1-2”, respectively. The leaves and fruits of these cultivars/strains are shown above the corresponding bands. Among these fruits, 1 and 4–6 are fully red, 7–9 are partly red, 3 and 10–11 are reddish, and 2 and 12–17 are fully green

The PCR amplification results by the primer pair F-2655 and R-2794, which encompassed the deleted region of the *PpBBX24* gene (Fig. 4a), showed that two bands with a size discrepancy of 14 bp were amplified from the DNA of “Zaosu Red” and 50 red F_1_ plants, and only the larger band was amplified from the DNA of “Zaosu”, “Kuala Pear”, and 50 green F_1_ plants (Fig. 4a). This revealed that the deletion exists only in the red plants.

The same *PpBBX24* gene fragments were also amplified using the DNA of other cultivars/strains whose young leaves and young fruits are of different colors, but the small band of 126 bp appeared only for the DNA of “Zaosu Red” (Fig. 4b). This revealed that the color mechanism of “Zaosu Red” was different from that of other red pears. Although both the young leaves and young fruits of “Zaosu Red”, “Red Anjou”, and “N3-1” are red, not all the tested cultivars/strains have the same color of young leaves and fruits (Fig. 4b). This further revealed that their color mechanisms are different.

**Fig. 5:**
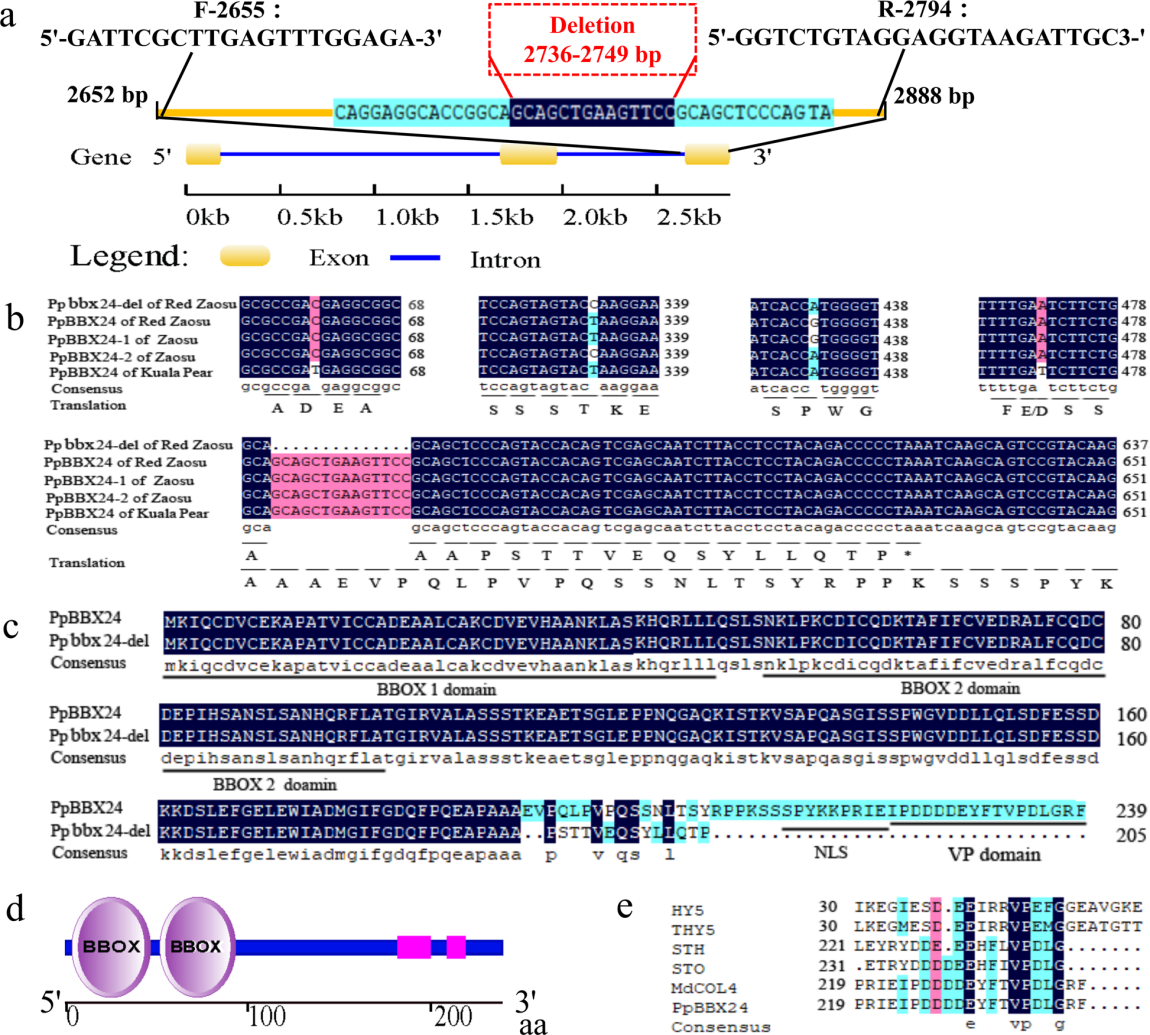
Bioinformatic analysis of the gene and protein sequences of PpBBX24 and its mutant. **a** Gene structure and deletion and primer locations of *PpBBX24*. The gene structure schematic was generated using GSDS 2.0 (http://gsds.cbi.pku.edu.cn/). The letters F and R represent the forward and reverse primers, respectively, and the numbers behind them represent the gene sequence order of the first nucleotide at the 5ʹ terminus of each primer. **b** CDS alignment and translation of *PpBBX24* and *Ppbbx24-del* in the three cultivars. **c** Protein sequence alignment of PpBBX24 and Ppbbx24-del in “Zaosu Red”. **d** Schematic of the conserved domains of PpBBX24, generated using SMART (http://smart.embl-heidelberg.de/). **e** Alignment of the VP domains of PpBB24, HY5 (GenBank: BAA21327.1), THY5 (GenBank: AJ011914.1), STO (GenBank: X95572), STH (GenBank: AF323666), and MdCOL4 (GenBank: ADL36673.1).

The full CDSs of the candidate *PpBBX24* gene both with and without the deletion were amplified from “Zaosu”, “Kuala Pear”, and “Zaosu Red”. The sequencing results showed that the full CDS without the deletion was 720 bp and encoded 239 aa. When 14 bp from 568 bp to 581 bp (from 2736 bp to 2749 bp in the gene sequence) were deleted, a coding frame shift and early termination occurred, and only 205 aa were translated (Fig. 5a–c). Aside from the 14 bp deletion, there were four other SNPs among the different types sequences of these three cultivars, which caused one amino acid alteration (Fig. 5b).

As Fig. 5a shows, this candidate gene has three exons and two introns, and the deletion is located in the last exon. The protein sequence contains two B-box domains at the N-terminus (Fig. 5d) belonging to the BBX family, also known as the COL transcription factor family, and was shown to have the highest homology with AtBBX24 (STO, AT1G06040.1) by BLASTP against the *Arabidopsis thaliana* Araport11 protein sequence database (https://www.arabidopsis.org/) and by phylogenetic analysis (Supplementary Fig. S2). Therefore, we named this gene and its mutant *PpBBX24* and *Ppbbx24-del*, respectively. Because of the early termination codon, a VP domain and a nuclear localization sequence (NLS) at the end of PpBBX24 are lacking in Ppbbx24-del (Fig. 5c). The VP domains of PpBBX24 are exactly the same as those of MdCOL4 and are homologous with those of HY5, THY5, STO, and STH (Fig. 5e).

### Transcription-level analysis of anthocyanin biosynthesis-related genes

**Fig. 6:**
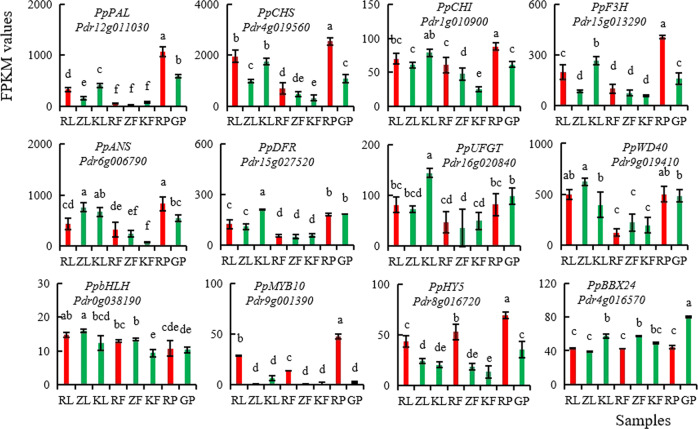
FPKM values of anthocyanin synthesis- and regulation-associated genes in the leaves and fruit peels of different pear cultivars according to RNA-seq. RL, ZL, and KL and RF, ZF, and KF represent young leaves and peels of young fruits of “Zaosu Red”, “Zaosu”, and “Kuala Pear”, respectively; RP and GP represent mixed leaves of the red pool and green pool, which were randomly collected from ten red and ten green hybrid progeny of “Kuala Pear” × “Zaosu Red” (three biological replicates of each pool were collected from 30 different plants), respectively. All the data are the means of three biological replications, with the error bars indicating ±SDs. The different lowercase letters above the bars are significantly different at *P* ≤ 0.05 (Duncan’s multiple range test). The column color is consistent with the sample color

Using RNA-seq, we obtained a total of 176 Gb clean data (590 M clean reads) from 24 pear samples (Supplementary Table S9), which have been archived at the NCBI SRA under accession SRP225824. According to the fragments per kilobase of transcript per million fragments mapped (FPKM) data, the transcript levels of genes encoding seven key anthocyanin synthesis enzymes, four important regulatory genes, and the *PpBBX24* gene were compared in the young leaves and peels of young fruits of “Zaosu Red”, “Zaosu”, and “Kuala Pear” and in the leaves of the red pool and green pool. The results showed that the transcript levels of the seven anthocyanin synthesis enzyme-encoding genes in the young leaves and peels of young fruits of “Zaosu Red” were not significantly higher than those of “Zaosu” and “Kuala Pear”, but the transcript levels of *PpPAL*, *PpCHS*, *PpCHI*, *PpF3H*, and *PpANS* in the young leaves of the red pool were significantly higher than those of the green pool. The transcript levels of the *PpMYB10* and *PpHY5* genes in all the tested red samples were significantly higher than those in the green samples. The *PpBBX24* gene presented slightly lower transcript levels in the red samples than in the green samples (Fig. 6).

## Discussion

### Differentially sourced red-colored pear mutants have various types of mutations

Mutation selection is one of the most efficient approaches to obtain red-skinned pear cultivars, and many red-skinned pear cultivars have been successfully selected in practice, such as “Red Anjou”^28^, “Red Comice”, “Royal Red Hardy”^9^, “Max Red Bartlett”^10^, “Bon Rouge”^29^, “Nanhong Li”^30^, and “Red Zaosu”^24^. However, which loci are mutated in the genome causing a green pear cultivar to turn red is rarely reported. In this study, we identified a 14 bp deletion in chromosome 4 of the red pear mutant “Zaosu Red” by combining BSA with whole-genome sequencing (Table 2). Using PCR amplification and sequencing, we determined that the deletion was present only in “Zaosu Red” and in its red F_1_ plants; thus, the deletion is closely linked to the red characteristic of the red pear mutant. However, no such deletion was found in the other tested pears, regardless of their phenotype, even despite “Red Anjou” also being red mutant and having the same phenotype as “Zaosu Red” (Fig. 4). This implies that the color mechanism is complex and varies among different red pear types.

Generally, compared with red plants or red young leaves, red fruit color is the characteristic we most expect. According to the phenotypes of the tested pear cultivars/accessions in this study, it was shown that a pear that produces red young leaves is not always able to produce red fruits. For example, “Huangguan” and “E1-2”, produce red young leaves but green fruits; other cultivars, such as “Red Comice” and “Zhongaihongli”, produce red fruits but have green leaves (Fig. 4b). The “Zaosu Red” pear we used for DNA-seq in this study not only produces red fruits and red young leaves but also produces red flowers, red stems, and red F_1_ plants (Fig. 1). Because the F_1_ plants we used in this study were small seedlings without fruits, we did not know if the red plants could also produce red fruits. According to the results of our anthocyanin content analysis (Fig. 2), the anthocyanin components between the young leaves and peels of young fruits of “Zaosu Red” were nearly the same, but the proportions of the main components were different, which revealed that the color mechanisms in the leaves and fruits may have both similarities and differences. However, whether the red color of both the plants and fruits is caused by the 14 bp deletion of the *PpBBX24* gene needs further study.

### Structure and function of the candidate *PpBBX24* gene

In this study, the candidate PpBBX24 associated with the red characteristic of “Zaosu Red” is a B-box protein and belongs to a class of zinc finger transcription factors that contain one or more B-box domains at the N-terminus involved in mediating protein–protein interactions, of which 32 members have been identified in *Arabidopsis*^31,32^. It has been verified that several plant BBXs play important regulatory roles in photomorphogenesis and anthocyanin accumulation, among which AtBBX20^33^, MdBBX20^34^, AtBBX21^35,36^, PpBBX16^6^, PpBBX18^25^, OsBBX14^37,38^, and MdBBX22^39,40^ act as positive regulators, whereas AtBBX19^41^, PpBBX21^23^, AtBBX32^42^, MdBBX37^43^, MdCOL4 (BBX24)^44^, AtBBX24^45^, and AtBBX25^35,46^ act as negative regulators.

PpBBX24 shares high homology with MdCOL4^44^ and AtBBX24^45^ (96.23% and 62.00%, respectively). The function of MdCOL4 and AtBBX24 would be a valuable clue for PpBBX24. In apple, MdCOL4 interacts with MdHY5 via the B-box2 domain of MdCOL4 and the bZIP domain of MdHY5 to synergistically inhibit the expression of *MdMYB1*. MdCOL4 also directly binds to the three G-box motifs in the promoters of *MdANS* and *MdUFGT* to suppress their expression^44^. AtBBX24 attenuates UV-B-induced HY5 accumulation and suppresses its transcriptional activation activity^45^, which is similar to that which occurs for MdCOL4. Compared with PpBBX24, Ppbbx24-del has 48 fewer aa at the C-terminus (Fig. 5b, c), so we predicted that the mutated Ppbbx24-del may cause its repressive function on the transcriptional activation activity of HY5 and the reduced expression of *MYB1/10*. Furthermore, premature Ppbbx24-del may lose its binding activity with the promoters of the *ANS* and *UFGT* genes and decrease the repression of gene expression. In this study, the *PpMYB10* and *PpHY5* genes were significantly upregulated in all the tested red samples compared with the green samples (Fig. 6), while the expression of the normal *PpBBX24* gene (full type) was reversed, which would essentially support the above assumptions. In addition, studying whether PpBBX24 could regulate the transcript level of the *PpHY5* gene directly would be an interesting future topic.

Previous studies have shown that AtBBX24 (STO) and AtBBX25 (STH) contain a reserved VP domain (Fig. 5e), which is necessary to interact with the WD40 domain of AtCOP1^47,48^. AtCOP1 is an E3 ubiquitin ligase that is mainly localized to the nucleus, and its physical interaction with AtBBX24 is required for the ubiquitination and degradation of BBX24 in the light^48^. Due to the 14 nucleotide deletion, two motifs, the NLS and VP domain, were lost from Ppbbx24-del (Fig. 5c), which may cause Ppbbx24-del to fail to localize to the nucleus and interact with COP1 and to not be subsequently ubiquitinated and degraded, resulting in the opposite activity of that of MYB1/10 and HY5 by the newly generated amino acids. However, all of the above hypotheses need to be studied further.

### The transcript level of the *PpBBX24* gene in red samples is relatively low

In this study, the *PpBBX24* gene also presented a lower transcript level in the red samples than in the green samples, but compared with the green samples, not all the red samples reached a significant level in terms of their *PpBBX24* transcript level. The *PpBBX24* gene in the red samples comprised both full and del types, while in the reference genome, the green samples represent only the full type. When the FPKM value was calculated, some of the del-type fragments failed to contribute to it, so the transcript levels (FPKM values) of the *PpBBX24* gene in the red samples here were slightly lower than those of the real sum of full and del types. We also amplified the *PpBBX24* gene using the cDNA of young leaves from “Zaosu Red” by PCR and sequenced the products at random. Among the 68 sequenced clones, 29 (42.65%) were full type, and 39 (57.35%) were del type. This implied that the transcript levels of the normal *PpBBX24* gene in the red samples were significantly lower than those in the green samples, which may greatly reduce the negative regulatory effect of this gene on photomorphogenesis and anthocyanin accumulation. This may be the key season why the color of “Zaosu Red” becomes red. However, whether the expression differences of the *MYB10* and *HY5* transcriptional factors were caused by the mutated Ppbbx24-del and its underlying regulatory mechanism need to be studied further.

### The combination of BSA and whole-genome sequencing is effective for the identification of causal genes

Along with the development of sequencing technology and the successful assembly of high-quality genomes, the combination of BSA and whole-genome sequencing has become a very quick and efficient approach for identifying causal genes, of which many have been successfully identified in many plant species^49–51^. To obtain ideal results, single plants of mixed pools are generally selected from F_2_ or backcross populations. Nevertheless, many fruit trees species, such as pear and apple, are limited by a long juvenile phase or self-incompatibility, making it hard to obtain F_2_ or backcross populations. Therefore, a few F_1_ populations were also used for BSA, but the target trait in the dominant parent must be heterozygous. It is also hard to obtain an accurate associated locus corresponding to the target trait. For example, Lu et al.^52^ located a dominant gene (temperature-sensitive semidwarf, Tssd) at an interval of 500 kb and obtained 69 candidate genes using an F_1_ population of peach. Xue et al.^27^ used an F_1_ pear population of “PremP109” × “Red Zaosu” to map the red foliage and fruit skin traits in a 368.6 kb region of LG4. In this study, we used an F_1_ pear population of “Kuala Pear” × “Zaosu Red” to identify the variants associated with the red color trait. The red parent has the same genetic background as that of the genotype that Xue et al.^27^ used. We also obtained a long associated region in chromosome 4; this region is located on the same chromosome and in the same neighboring region as that reported by Xue et al.^27^ and contains hundreds of predicted protein-coding genes (Table 1). It is fortunate that the red parent is a mutant of a green cultivar. We combined the associated analysis with the differential analysis of the red parent and its original type and ultimately identified the key associated locus and gene.

In this study, the final candidate variant was located at 18,388,296 bp of Chr 4 and was closer to the highest associated peak region (17.3 Mb) calculated by the ED method than by the SNP index method (8.2 Mb), which means that the ED method may be more suitable for highly heterozygous species such as pear in the same associated analysis. However, if the target characteristic is not sourced from a mutation, it would be very hard work or even impossible to find the associated locus accurately by this method. Therefore, we strongly suggest that when an F_1_ population is used for BSA, including the mutant as one of the parents is the best choice.

In conclusion, a deletion in the coding region of *PpBBX24* was identified from the red skin of “Zaosu Red” pear and may be the key factor that causes “Zaosu Red” to turn red. This study will provide new perspectives for the study of plant anthocyanin regulation and red pear breeding.

## Materials and methods

### Plant materials

A cross of “Kuala Pear” (male parent, a local cultivar of *P. sinkiangensis* grown in China, Fig. 1g, j) and “Zaosu Red” (male parent, Fig. 1f, i) was performed in 2016, and more than 1000 F_1_ plants were obtained in 2017, out of which red and green plants were produced at ~1:1 (Fig. 1k). In this study, the young leaves of 50 red F_1_ plants and 50 green F_1_ plants selected at random from the above cross were collected to construct red and green gene pools for BSA, the young leaves of 30 red F_1_ plants and 30 green F_1_ plants (10 of which were mixed for biological replication) were collected for RNA-seq, and last, the young leaves and peels of young fruits of “Kuala Pear”, “Zaosu Red”, and “Zaosu” were collected for anthocyanin content measurement, DNA-seq, RNA-seq, and gene cloning. Three biological replications were included for anthocyanin content measurement and RNA-seq. All the trial plants were grown in the orchard of the Horticultural Crops Research Institute, Xinjiang Academy of Agricultural Sciences (Luntai, China, 41°47ʹ4ʺ N, 84°14ʹ10ʺ E) and sampled from 2017 to 2019.

### Extraction and measurement of anthocyanins

The extraction of anthocyanins was performed according to the protocol described by Yao et al.^23^ but was slightly modified as follows. Approximately 0.2 g of pear peels was ground in liquid nitrogen and suspended in methanol consisting of 0.1% HCl in the dark for 24 h. Afterward, 2 ml of the supernatant was then pipetted into a new tube and evaporated using a rotary evaporator (R-210, Buch, Switzerland) under vacuum at 40 °C. The residue was resuspended in acidified water (1.18 mM HCl) and then extracted with a preconditioned C18 solid-phase column (Oasis HLB, 30 μm, Waters, Eschborn, Germany). The anthocyanins were ultimately eluted with 1 ml of methanol and filtered through a 0.22 μm Millipore membrane (Millipore, Merck, USA). The anthocyanins were subsequently analyzed using a Waters Acquity UPLC system coupled to an Acquity PDA eλ diode array detector and a Xevo TQD mass spectrometer (Waters, MA, USA) equipped with an Acquity UPLC HSS T3 column (2.1 mm × 150 mm, 1.8 μm particle size, Waters, Eschborn, Germany). In brief, 2 μl of the filtered sample was injected and run at 40 °C (column temperature) and 0.3 ml/min (flow rate) using mobile phases A (methanol:acetonitrile, 7:3, v/v) and B (formic acid:water, 5:95, v/v). The linear gradient of phase A was 10% for the first 2 min, after which it increased from 10 to 25% from 2 min to 30 min, increased from 25 to 40% from 30 to 60 min, increased from 40 to 80% from 60 to 61 min, and then decreased from 80 to 10% from 61 to 62 min. Finally, an isocratic elution with 10% phase A was maintained for 13 min. The MS/MS parameters were as follows: ESI source of positive ion mode, MRM model scanning, source temperature of 150 °C, probe temperature of 500 °C, desolvation gas flow of 800 L/h, cone gas flow of 50 L/h, and collision gas (high-purity argon) flow of 0.13 ml/min. The PDA detector wavelength was set to 520 nm. The standard samples of cyanidin-3-O-glucoside, cyanidin-3-O-arabinoside, and peonidin-3-O-galactoside were obtained from Sigma-Aldrich (MO, USA), and C-Ga was obtained from PhytoLab (Vestenbergsgreuth, Germany).

### DNA extraction and whole-genome sequencing

Genomic DNA was extracted from fresh leaves of 50 red F_1_ plants and 50 green F_1_ plants as well as their two parents and “Zaosu” using the cetyl-trimethylammonium bromide method^53^ and subsequently quantified using a NanoDrop 2000 spectrophotometer (Thermo Scientific, MA, USA). Equal amounts of DNA from 50 red plants and 50 green plants were then mixed to construct the red pools and green pools. The DNA of the two parents, two pools, and “Zaosu” was sonicated to generate 350 bp fragments for the construction of DNA sequencing libraries using a TruSeq DNA Sample Preparation Kit (Illumina, CA, USA) following the manufacturer’s recommendations. The qualified libraries were sequenced at Biomarker Technologies Corporation (Beijing, China) on an Illumina HiSeq X-Ten platform (Illumina, CA, USA).

### Read alignment, SNP/InDel variant detection, and annotation

The raw data were qualified, and the adapter sequences and low-quality reads (proportion of noncalled bases >5%) were removed. High-quality clean reads were aligned to the pear genome (GCA_008932095.1)^54^ using BWA software with the default parameters^55^. According to the location results of the clean reads in the reference genome, the duplicated reads were filtered using Picard (http://broadinstitute.github.io/picard/). The SNP/InDel variants were then detected and filtered using the Genome Analysis Toolkit^56^ (GATK, https://software.broadinstitute.org/gatk/) and annotated using SnpEff^57^, which included determining their locations in the genome and their effects on genes.

### Identification and analysis of trait-associated SNPs/InDels

The SNPs/InDels between the two parents and between the two pools were extracted from their vcf files. Two methods, the ED^58^, and the SNP index^59^, were used to assess the association of these SNPs/InDels with the target characteristics based on their read depth information in the red pool and green pool. The ED value was calculated as follows:

$${\mathrm{ED}} = \sqrt {\left( {A{\mathrm{red}} - A{\mathrm{green}}} \right)^2 + \left( {G{\mathrm{red}} - {G{\mathrm{green}}}} \right)^2 + \left( {C{\mathrm{red}} - {C{\mathrm{green}}}} \right)^2 + \left( {T{\mathrm{red}} - {T{\mathrm{green}}}} \right)^2}$$

in which each *A*, *G*, *C*, and *T* letter represents the frequency of its corresponding DNA nucleotide in the red pool and green pool, and the higher the ED value is, the stronger the variant associated with the target characteristic. The SNP index value was calculated as follows:

$${\mathrm{SNP}}\;{\mathrm{index}}\left( {{\mathrm{aa}}} \right) = {{M{\mathrm{aa}}/}}\left({{M{\mathrm{aa}}} + {P{\mathrm{aa}}}} \right)$$

$${\mathrm{SNP}}\;{\mathrm{index}}\left( {{\mathrm{ab}}} \right) = {M{\mathrm{ab}}/}\left( {M{\mathrm{ab}} + {P{\mathrm{ab}}}} \right)$$

$$\Delta {\mathrm{SNP}}\;{\mathrm{index}} = {\mathrm{SNP}}\;{\mathrm{index}}\left( {{\mathrm{aa}}} \right) - {\mathrm{SNP}}\;{\mathrm{index}}\left( {{\mathrm{ab}}} \right)$$

in which aa and ab represent the green pool and red pool, respectively, and *M* and *P* represent the frequency of the variant from the female and male parents, respectively; the closer to 1 the ΔSNP index value is, the stronger the variant associated with the target characteristic. To eliminate background noise and false-positive sites, the fifth power of the original ED value was taken as the association value^58^. The fifth power of the ED and the ΔSNP index values from the same chromosome were then fitted using a sliding window approach with 2 Mb windows sliding in 10 kb steps. Finally, the regions whose fitted ED and ΔSNP index values were over the theoretical thresholds were selected as candidate regions.

The SNPs/InDels between “Zaosu” and “Zaosu Red” in the candidate regions were also extracted, and the mutual SNPs/InDels between the two parents, the two pools, “Zaosu”, and “Zaosu Red” in the candidate regions were further obtained. The mutual SNPs/InDels in the candidate regions, which were homozygous in the green parent and pool and heterozygous in the red parent and pool, were chosen as the final variants. According to the annotated information, the genes that contain or were near the final variants were chosen as candidate genes associated with the mutated trait for further analysis.

### Genotypic identification

To verify the association of a selected InDel with the red trait of “Zaosu Red”, we designed a pair of primers that encompasses the deleted region (F-2655: 5ʹ-GATTCGCTTGAGTTTGGAGA-3ʹ and R-2794: 5ʹ-GGTCTGTAGGAGGTAAGATTGC-3ʹ) according to the sequence information. PCR amplification was then performed using this pair of primers on the DNA samples of the two parents, “Zaosu”, 50 red F_1_ plants, 50 green F_1_ single plants, and other cultivars/accessions. The PCR products were examined using a DNA 1K Chip (PN 760517) & Reagent Kit (PN CLS 760673) on a LabChip GX Touch HT Nucleic Acid Analyzer (Perkin-Elmer, Connecticut, USA).

### Cloning and bioinformatic analysis of the *PpBBX24* gene

The total RNA of “Zaosu”, “Zaosu Red”, and “Kuala Pear” was extracted from young leaves using an EasySpin Plus Plant RNA Kit (RN38, Aidlab, Beijing, China), and first-strand cDNA was synthesized from 2 μg of DNA-free RNA using a PrimeScript First-Strand cDNA Synthesis Kit (6110A, TaKaRa, Ohtsu, Japan) following the manufacturer’s instructions. The cDNA was diluted fivefold for use in gene cloning. The CDSs of the *PpBBX24* (with the deleted region) and *Ppbbx24-del* (without the deleted region) genes were amplified using PrimeSTAR GXL DNA Polymerase (R050Q, TaKaRa, Ohtsu, Japan) with the primer pair ColF (5ʹ-ATGAAGATTCAGTGTGATGTGTGC-3ʹ) and ColR (5ʹ-CTAAAATCTGCCGAGGTCAGG-3ʹ) and then sequenced by Taihe Biotechnology Co., Ltd (Beijing, China). The cloned sequences were subsequently translated and aligned using DNAMAN 6 software. The conserved domains were searched using the online software SMART (http://smart.embl-heidelberg.de/), and the gene structure was analyzed using the Gene Structure Display Server 2.0 (http://gsds.cbi.pku.edu.cn/).

### RNA isolation, library preparation, and RNA-seq

Total RNA was isolated using TRIzol reagent (Invitrogen, San Diego, USA) following the manufacturer’s protocol and then purified using oligo (dT) magnetic beads. The integrity of the RNA was verified with an Agilent 2100 Bioanalyzer (Agilent Technologies, Palo Alto, USA). The paired-end library was prepared using a TruSeq RNA Sample Preparation kit (Illumina, San Diego, USA), and the libraries were sequenced by Biomarker Technology Company (Beijing, China) on an Illumina HiSeq X-Ten platform (Illumina, CA, USA).

### **G**ene transcription analysis

After filtering, the clean reads were aligned against the reference genome (GCA_008932095.1)^53^ using HISAT2^60^ and StringTie^61^ software. The FPKM value^62^ of every gene was then calculated to estimate the transcript levels of every sample using the following formula:

$${\rm FPKM} = \frac{{\rm cDNA}\;{\rm fragments}}{{\rm Mapped}\;{\rm fragments}\;{(\rm millions)} \times \;{\rm Transcript}\;{\rm length}\;{(\rm kb)}}$$

## Supplementary information

Supplementary figures and tables

## Data Availability

The data associated with this study have been deposited in NCBI under accession number PRJNA577323. The raw data from this project have been deposited in the NCBI SRA database under accession number SRP225824.
