# Supplementary material for: A 14 nucleotide deletion mutation in the coding region of the PpBBX24 gene is associated with the red skin of “Zaosu Red” pear (Pyrus pyrifolia White Pear Group): a deletion in the PpBBX24 gene is associated with the red skin of pear
Source: Hortic Res. 2020 Apr 1;7:39. doi: 10.1038/s41438-020-0259-7 (PMC7109114; doi:10.1038/s41438-020-0259-7)
Supplement: Supplementary file 1 — Supplementary figures and tables [file 41438_2020_259_MOESM1_ESM.docx]

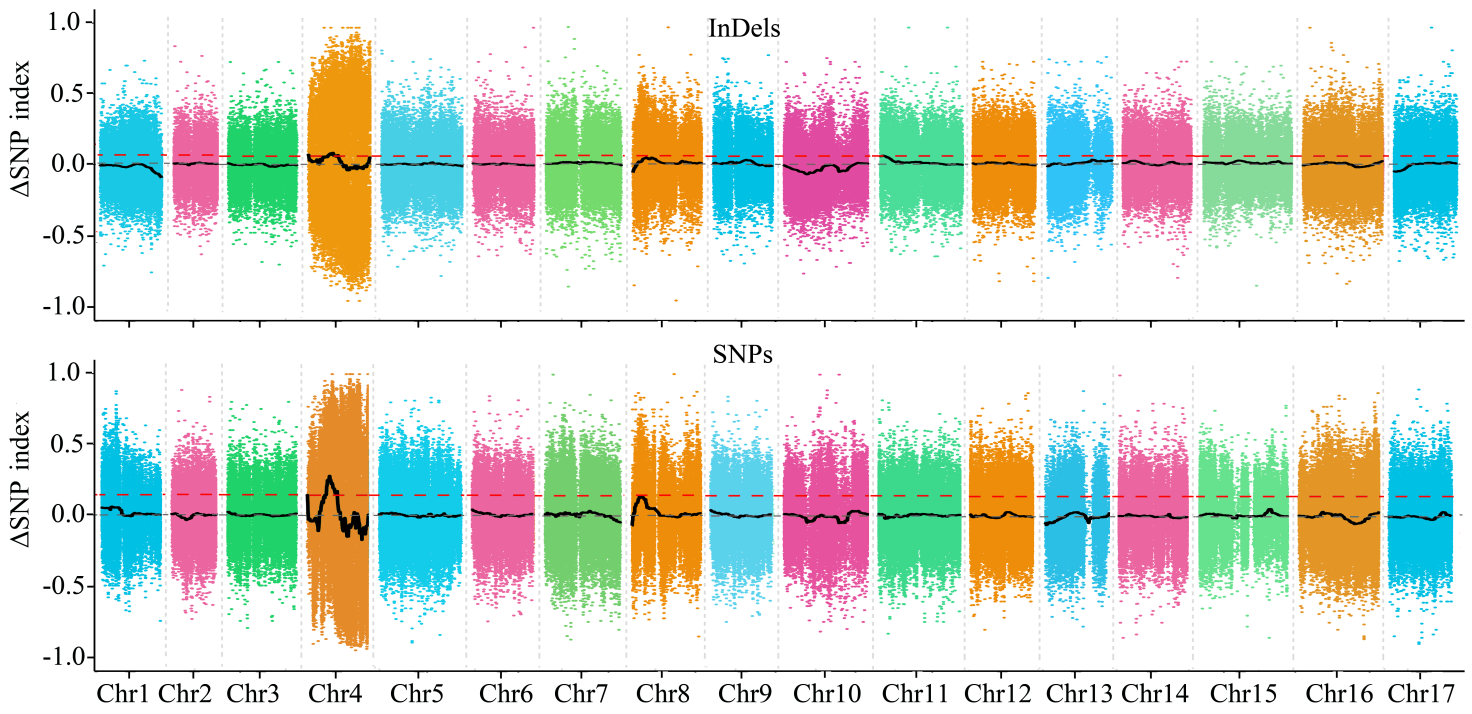


**Supplementary** **Fig. S1 ΔSNP index value distribution of InDels and SNPs in chromosomes.** The abscissa represents the chromosome name, colored dots represent ΔSNP index values, the black lines represent the fitted ΔSNP index values (with 2 Mb windows sliding in 10 kb steps), and the red dotted lines represent the thresholds of 99 percentiles.

**Supplementary** **Fig. S2**  Phylogenetic tree constructed using PpBBX24, MdCOL4 and 32 *Arabidopsis thaliana* BBX transcription factors by MEGA5.

**Supplementary Table S1 Summary of DNA sequencing and mapping.**

| Samples | Number of clean reads (M) | Number of clean base (Gb) | Ratio of Q30 base (%) | Ratio of GC  (%) | Sequencing depth  (×) | Mapped ratio  (%) | Properly mapped ratio (%) |
| --- | --- | --- | --- | --- | --- | --- | --- |
| Zaosu Red | 43.99 | 13.18 | 92.81 | 37.67 | 25.78 | 92.09 | 81.08 |
| Zaosu | 39.14 | 11.73 | 95.02 | 38.23 | 25.21 | 95.38 | 80.21 |
| Kuala Pear | 95.18 | 28.52 | 92.49 | 37.59 | 22.93 | 90.72 | 80.8 |
| Green pool | 102.12 | 30.60 | 93.16 | 37.59 | 55.77 | 91.09 | 79.75 |
| Red pool | 43.11 | 12.89 | 93.01 | 37.7 | 59.84 | 91.53 | 80.76 |

**Supplementary Table S2 Summary of detected SNPs and InDels in different samples.**

| Variants | Samples | Total (M) | Transition (M) | Transversion (M) | Transition/ Transversion | Heterozygosity (M) | Homozygosity (M) | Heterozygosity ratio (%) |
| --- | --- | --- | --- | --- | --- | --- | --- | --- |
| SNPs | Red Zaosu | 5.08 | 3.45 | 1.63 | 2.11 | 3.31 | 1.76 | 65.25 |
|  | Zaosu | 5.53 | 3.77 | 1.76 | 2.14 | 3.49 | 2.04 | 63.08 |
|  | Kuala Pear | 4.86 | 3.30 | 1.57 | 2.10 | 2.94 | 1.92 | 60.46 |
|  | Green pool | 6.89 | 4.68 | 2.21 | 2.11 | 5.67 | 1.22 | 82.24 |
|  | Red pool | 6.90 | 4.69 | 2.21 | 2.12 | 5.68 | 1.22 | 82.28 |
| InDels | Red Zaosu | 0.95 | 0.46 | 0.49 | 0.94 | 0.29 | 0.65 | 31.10 |
|  | Zaosu | 0.98 | 0.48 | 0.50 | 0.96 | 0.32 | 0.66 | 32.72 |
|  | Kuala Pear | 0.92 | 0.44 | 0.47 | 0.94 | 0.33 | 0.58 | 36.35 |
|  | Green pool | 1.26 | 0.60 | 0.67 | 0.89 | 0.19 | 1.08 | 14.71 |
|  | Red pool | 1.27 | 0.60 | 0.67 | 0.89 | 0.19 | 1.08 | 14.69 |

**Supplementary Table S3 Summary of SNPs annotation in different samples.**

| Annotation | Zaosu Red | | Zaosu | | Kuala Pear | | Green pool | | Red pool | |
| --- | --- | --- | --- | --- | --- | --- | --- | --- | --- | --- |
|  | Number | % | Number | % | Number | % | Number | % | Number | % |
| CDS | 323,824 | 6.38 | 341,729 | 6.17 | 317,846 | 6.53 | 416,997 | 6.05 | 416,346 | 6.04 |
| Splice site donor | 333 | 0.01 | 335 | 0.01 | 327 | 0.01 | 464 | 0.01 | 455 | 0.01 |
| Intergenic | 1,724,554 | 33.96 | 1,922,746 | 34.74 | 1,615,186 | 33.21 | 2,356,268 | 34.19 | 2,362,725 | 34.25 |
| Splice site acceptor | 345 | 0.01 | 367 | 0.01 | 334 | 0.01 | 485 | 0.01 | 481 | 0.01 |
| Upstream | 1,131,846 | 22.29 | 1,226,399 | 22.16 | 1,090,698 | 22.42 | 1,541,339 | 22.36 | 1,540,937 | 22.34 |
| Intragenic | 2,595 | 0.05 | 2,809 | 0.05 | 2,429 | 0.05 | 3,469 | 0.05 | 3,447 | 0.05 |
| Downstream | 951,925 | 18.74 | 1,030,047 | 18.61 | 918,072 | 18.87 | 1,295,964 | 18.80 | 1,296,375 | 18.79 |
| Start gained | 6,132 | 0.12 | 6,430 | 0.12 | 6,050 | 0.12 | 8,380 | 0.12 | 8,371 | 0.12 |
| Intron | 475,350 | 9.36 | 506,736 | 9.16 | 467,764 | 9.62 | 646,085 | 9.37 | 645,419 | 9.36 |
| Splice site region | 13,910 | 0.27 | 14,720 | 0.27 | 13,626 | 0.28 | 18,050 | 0.26 | 18,059 | 0.26 |
| Other | 325,192 | 6.40 | 352,398 | 6.37 | 36,891 | 0.76 | 438,518 | 6.36 | 439,541 | 6.37 |
| UTR 5 primer | 37,726 | 0.74 | 40,070 | 0.72 | 311,495 | 6.40 | 50,700 | 0.74 | 50,610 | 0.73 |
| UTR 3 primer | 84,713 | 1.67 | 90,006 | 1.63 | 83,250 | 1.71 | 115,253 | 1.67 | 115,126 | 1.67 |
| total | 5,078,445 | 100 | 5,534,792 | 100 | 4,863,968 | 100 | 6,891,972 | 100 | 6,897,892 | 100 |

**Supplementary Table S4 Summary of SNPs annotation in CDS regions in different samples.**

| Annotation | Zaosu Red | | Zaosu | | Kuala Pear | | Green pool | | Red pool | |
| --- | --- | --- | --- | --- | --- | --- | --- | --- | --- | --- |
|  | Number | % | Number | % | Number | % | Number | % | Number | % |
| Non synonymous start | 40 | 0.01 | 47 | 0.01 | 28 | 0.01 | 47 | 0.01 | 50 | 0.01 |
| Synonymous coding | 174,434 | 53.87 | 182,730 | 53.47 | 171,598 | 53.99 | 220,887 | 52.97 | 220,462 | 52.95 |
| Start lost | 224 | 0.07 | 250 | 0.07 | 209 | 0.07 | 316 | 0.08 | 309 | 0.07 |
| Non-synonymous coding | 147,047 | 45.41 | 156,394 | 45.77 | 143,988 | 45.30 | 192,800 | 46.24 | 192,578 | 46.25 |
| Synonymous stop | 251 | 0.08 | 265 | 0.08 | 248 | 0.08 | 344 | 0.08 | 345 | 0.08 |
| Stop gained | 1,493 | 0.46 | 1,679 | 0.49 | 1,437 | 0.45 | 2,171 | 0.52 | 2,166 | 0.52 |
| Stop lost | 335 | 0.10 | 364 | 0.11 | 338 | 0.11 | 432 | 0.10 | 436 | 0.10 |
| Total | 323,824 | 100 | 341,729 | 100 | 317,846 | 100 | 416,997 | 100 | 416,346 | 100 |

**Supplementary Table S5 Summary of InDels annotation in different samples.**

| Annotation | Zaosu Red | | Kuala Pear | | Green pool | | Red pool | | Zaosu | |
| --- | --- | --- | --- | --- | --- | --- | --- | --- | --- | --- |
|  | Number | % | Number | % | Number | % | Number | % | Number | % |
| CDS | 14,783 | 1.56 | 14,646 | 1.60 | 18,846 | 1.49 | 18,853 | 1.49 | 16,385 | 1.67 |
| UTR 3 deleted | 0 | 0.00 | 1 | 0.00 | 1 | 0.00 | 1 | 0.00 | 0 | 0.00 |
| Intergenic | 278,417 | 29.45 | 265,031 | 28.89 | 377,910 | 29.88 | 379,398 | 29.96 | 286,089 | 29.10 |
| Splice site acceptor | 209 | 0.02 | 187 | 0.02 | 247 | 0.02 | 245 | 0.02 | 208 | 0.02 |
| Upstream | 240,358 | 25.43 | 233,985 | 25.51 | 323,189 | 25.56 | 323,328 | 25.53 | 247,461 | 25.17 |
| Splice site donor | 342 | 0.04 | 325 | 0.04 | 424 | 0.03 | 427 | 0.03 | 364 | 0.04 |
| Downstream | 193,421 | 20.46 | 188,606 | 20.56 | 259,979 | 20.56 | 260,114 | 20.54 | 198,974 | 20.24 |
| Intragenic | 910 | 0.10 | 959 | 0.10 | 1,258 | 0.10 | 1,258 | 0.10 | 958 | 0.10 |
| Intron | 122,100 | 12.92 | 120,912 | 13.18 | 158,172 | 12.51 | 157,914 | 12.47 | 131,773 | 13.40 |
| Splice site region | 3,129 | 0.33 | 3,075 | 0.34 | 3,738 | 0.30 | 3,769 | 0.30 | 3,408 | 0.35 |
| Other | 56,377 | 5.96 | 54,725 | 5.97 | 74,650 | 5.90 | 74,787 | 5.91 | 59,071 | 6.01 |
| UTR 3 primer | 20,316 | 2.15 | 20,075 | 2.19 | 26,704 | 2.11 | 26,766 | 2.11 | 22,125 | 2.25 |
| UTR 5 primer | 14,925 | 1.58 | 14,856 | 1.62 | 19,434 | 1.54 | 19,467 | 1.54 | 16,248 | 1.65 |
| Total | 945,287 | 100 | 917,383 | 100 | 1,264,552 | 100 | 1,266,327 | 100 | 983064 | 100 |

**Supplementary Table S6 Summary of InDels annotation in CDS regions in different samples.**

| Annotation | Zaosu Red | | Kuala Pear | | Green pool | | Red pool | | Zaosu | |
| --- | --- | --- | --- | --- | --- | --- | --- | --- | --- | --- |
|  | Number | % | Number | % | Number | % | Number | % | Number | % |
| Exon deleted | 2 | 0.01 | 1 | 0.01 | 1 | 0.01 | 2 | 0.01 | 0 | 0.00 |
| Frame shift | 7,999 | 54.11 | 8,009 | 54.68 | 10,276 | 54.53 | 10,263 | 54.44 | 9,051 | 55.24 |
| Stop lost | 83 | 0.56 | 96 | 0.66 | 119 | 0.63 | 122 | 0.65 | 115 | 0.70 |
| Codon insertion | 2,292 | 15.50 | 2,209 | 15.08 | 2,778 | 14.74 | 2,794 | 14.82 | 2,436 | 14.87 |
| Start lost | 134 | 0.91 | 127 | 0.87 | 172 | 0.91 | 169 | 0.90 | 87 | 0.53 |
| Coding deletion | 1,901 | 12.86 | 1,854 | 12.66 | 2,489 | 13.21 | 2,488 | 13.20 | 2,016 | 12.30 |
| Stop gained | 170 | 1.15 | 143 | 0.98 | 195 | 1.03 | 196 | 1.04 | 181 | 1.10 |
| Codon change plus codon deletion | 1,297 | 8.77 | 1,308 | 8.93 | 1,728 | 9.17 | 1,729 | 9.17 | 1,472 | 8.98 |
| Codon change plus codon insertion | 905 | 6.12 | 899 | 6.14 | 1,088 | 5.77 | 1,090 | 5.78 | 1,027 | 6.27 |
| Total | 14,783 | 100 | 14,646 | 100 | 18,846 | 100 | 18,853 | 100 | 16,385 | 100.00 |

**Supplementary Table S7 Summary of the variant filteration.**

| Variant type | Total | Locus with multiple gene types | Locus supported by less than four reads | Locus with same gene type in both pools | Locus filtered by parents | High quality variants |
| --- | --- | --- | --- | --- | --- | --- |
| SNP | 7,218,542 | 67,789 | 181,707 | 1,153,130 | 5,084,035 | 731,881 |
| InDel | 1,326,611 | 98,820 | 47,403 | 160,631 | 549,264 | 470,493 |

**Supplementary Table S8 Screening of differential SNP/InDels in Chr 4.**

| Variant type | Zaosu Red vs Zaosu in Chr 4 | Zaosu Red vs Kuala Pear in the candidate associated region of Chr 4 | Intersection | Heterozygous in Red Zaosu/red pool and homozygous in Kuala Pear/Zaosu/green pool |
| --- | --- | --- | --- | --- |
| SNP | 19,141 | 7,470 | 252 | 5 |
| InDel | 3,172 | 4,686 | 150 | 8 |

**Supplementary Table S9 Summary of RNA sequencing and mapping.**

| **Cultivar/Pool** | **Organism** | **Repetition** | **Clean reads** | **Clean bases** | **%≥Q30** | **Mapped Reads %** |
| --- | --- | --- | --- | --- | --- | --- |
| Zaosu Red | Young leaves | 1 | 25,399,284 | 7,567,853,048 | 95.30 | 79.11 |
|  |  | 2 | 23,363,581 | 6,956,499,976 | 95.31 | 79.52 |
|  |  | 3 | 26,771,815 | 7,997,500,414 | 94.99 | 79.70 |
| Zaosu |  | 1 | 26,791,043 | 7,999,756,466 | 95.09 | 80.72 |
|  |  | 2 | 27,160,300 | 8,111,807,412 | 95.24 | 79.87 |
|  |  | 3 | 22,524,574 | 6,731,877,764 | 95.35 | 79.44 |
| Kuala Pear |  | 1 | 26,879,378 | 8,004,447,156 | 95.17 | 78.65 |
|  |  | 2 | 28,130,517 | 8,392,374,148 | 95.34 | 79.08 |
|  |  | 3 | 23,088,406 | 6,879,798,042 | 95.29 | 79.22 |
| Zaosu Red | Young fruit peels | 1 | 24,446,828 | 7,290,302,406 | 95.46 | 80.19 |
|  |  | 2 | 22,775,061 | 6,793,997,120 | 95.28 | 79.46 |
|  |  | 3 | 21,316,660 | 6,357,123,570 | 95.41 | 79.02 |
| Zaosu |  | 1 | 25,132,724 | 7,503,318,506 | 95.00 | 79.30 |
|  |  | 2 | 21,586,444 | 6,437,502,658 | 95.40 | 79.45 |
|  |  | 3 | 20,751,622 | 6,197,001,234 | 95.07 | 78.75 |
| Kuala Pear |  | 1 | 28,319,850 | 8,447,424,892 | 95.64 | 79.35 |
|  |  | 2 | 22,825,924 | 6,808,744,560 | 95.76 | 80.32 |
|  |  | 3 | 25,424,802 | 7,585,227,154 | 95.35 | 79.38 |
| Red pool | Young leaves | 1 | 25,277,546 | 7,540,987,212 | 95.17 | 80.90 |
|  |  | 2 | 26,046,667 | 7,768,022,556 | 95.33 | 80.51 |
|  |  | 3 | 24,074,392 | 7,182,739,840 | 95.25 | 80.03 |
| Green pool |  | 1 | 24,214,351 | 7,220,153,960 | 95.50 | 80.27 |
|  |  | 2 | 23,928,704 | 7,145,812,522 | 95.33 | 80.93 |
|  |  | 3 | 23,812,515 | 7,109,048,006 | 95.16 | 80.10 |
| Total | | | 590,042,988 | 176,029,320,622 | - | - |
